# Fuzzy Mobile-Robot Positioning in Intelligent Spaces Using Wireless Sensor Networks

**DOI:** 10.3390/s111110820

**Published:** 2011-11-17

**Authors:** David Herrero, Humberto Martínez

**Affiliations:** Department of Information and Communications Engineering, University of Murcia, 30100 Espinardo, Murcia, Spain; E-Mail: dherrero@um.es

**Keywords:** fuzzy logic, self-localization, autonomous robots, intelligent spaces, wireless sensor networks

## Abstract

This work presents the development and experimental evaluation of a method based on fuzzy logic to locate mobile robots in an Intelligent Space using Wireless Sensor Networks (WSNs). The problem consists of locating a mobile node using only inter-node range measurements, which are estimated by radio frequency signal strength attenuation. The sensor model of these measurements is very noisy and unreliable. The proposed method makes use of fuzzy logic for modeling and dealing with such uncertain information. Besides, the proposed approach is compared with a probabilistic technique showing that the fuzzy approach is able to handle highly uncertain situations that are difficult to manage by well-known localization methods.

## 1. Introduction

Nowadays, Wireless Sensor Networks (WSNs) [1] have gained an increasing attention thanks to the advances in wireless communications and sensor design, which have permitted to reduce the cost and size of sensor devices. These sensor networks are composed of autonomous wireless sensing devices that incorporate sensing, processing, storing, and communication capabilities. In order to classify them, there are diverse criteria in the literature, such as considering only the communication protocols [2], the nature of the specific application [3], and the wireless device functionalities [4]. They have been successfully applied in a wide spectrum of applications, such as search and rescue [5], disaster relief [6], target tracking [7], and smart environments [8], to name but a few.

The low cost of these devices makes them especially suitable for large Intelligent Spaces [9], where the nodes are spatially distributed in order to cooperatively processing and communicating sensed information. The positioning of mobile nodes along an Intelligent Space has special interest for location-dependent applications, such as robot navigation [10,11], geometric-dependent routing [12], location-dependent sensing, and Location-Based Services (LBS) [13].

The WSN localization problem consists of estimating the location or spatial coordinates of some or all the sensor network nodes of the WSN. In order to do so, the different localization approaches make assumptions about their network and device capabilities, including hardware incorporated in devices, signal propagation models, computational and energy requirements, nature of environment (indoor *vs.* outdoor), communication cost, accuracy requirements, and node mobility. Considering all these constraints, each sensor node makes use of available information, such as position measurements and location of neighbor nodes, to estimate its pose.

The localization problem is much more complex in indoors because Global Positioning System (GPS) coverage is limited and inter-node position measurements are usually unreliable in low-cost sensor devices. For these reasons, indoor WSN localization problem is usually simplified by differentiating between *unknown* and *known* sensor nodes. The former nodes make use of known location of latter ones, the so-called *beacons* or *anchor* nodes, and position measurements to estimate their location. The position measurements include both information about the sensor node position relative to the WSN, e.g., distance [14] or bearing [15] to *beacons*, and information on the sensor node motion, such as movement estimation obtained from accelerometers in sensor nodes [16] and from odometers in mobile robots [17].

What really makes indoor WSN localization difficult is the presence of uncertainty in position measurements and the reduced level of accuracy of beacon positioning. The sensor nodes make use of some signal propagation model, which should be calibrated for each specific environment, and hence, it is strongly affected by slight environmental modifications. Besides, the location of beacons is usually configured by hand in indoor applications, which gives rise to a reduced level of accuracy of beacon positioning. All these factors induce different types of uncertainty in position measurements, including vagueness, imprecision, unreliability, and random noise. Measurements may also be affected by several simultaneous factors, which are not necessarily independent.

For all these reasons it is important that the formalism used to address the indoor WSN localization problem is able to represent the different types of uncertainty and account for the differences between them. Fuzzy logic provides powerful tools to represent and handle the different facets of uncertainty in measurements [18], to address matching problems based on similarity interpretation of fuzzy logic [19], and to use approximate models based on experience. These arguments have induced us to make use of fuzzy sets as uncertainty representation of locations in the indoor WSN localization problem.

In this paper, we address the problem of positioning a mobile robot in an Intelligent Space [20–22] using a low-cost and low-density WSN composed of *TMote Sky* devices, which are equipped with ZigBee (IEEE 802.15.4) communications. The inter-node measurements are estimated using the Received Signal Strength (RSS) of Radio-Frequency (RF) communications. These measurements are really unreliable due to RF signal propagation effects, such as reflections, diffraction, and scattering, which make difficult the signal strength calibration. The robot makes use of a vague description of the environment and position measurements to estimate its pose. Thus, the restrictions of the problem are as follows: the knowledge of the environment is approximate, the density of the WSN is unknown, and the on-site startup cannot be complex or time consuming. We have adopted a fuzzy robot localization framework [23], based on early ideas for representing location uncertainty [24] and ambiguity [25] in position measurements, which combines the typical schema of fuzzy systems with the typical schema of recursive position estimation methods. The advantages of this approach are obvious when high uncertainty and sensor model ignorance, which are the typical conditions of indoor WSN applications using RSS for inter-node distance estimation.

The paper is structured as follows. Section 2 presents a review of relevant related works. Section 3 is devoted to analyze the sensor model used for estimating the distances between the sensor node installed on the robot platform and the beacons distributed along the Intelligent Space. Section 4 describes the theoretical bases of the proposed approach and a reference method used to evaluate the proposed one. The experimental setup, the experimental validation of the proposed method in different situations, and a comparison between the proposed approach and one of most popular localization methods is presented in Section 5. Finally, conclusions are presented in Section 6.

## 2. Related Works

Currently, there is a consensus on classification of WSN localization techniques into range-free (or coarse-grained) and range-based (or fine-grained) schemes [26–28]. *Range-free* approaches infer the constraints on the proximity to beacon nodes without making use of inter-node measurements, and thus sensing devices do not require special and expensive hardware. Normally, these localization methods use quite simple operations to save computational and energy consumption. They are used when the cost and limitation of hardware on sensing nodes prevent the use of range-based techniques, being a cost-effective alternative, at the cost of accuracy, in some applications [29]. On the other hand, *range-based* approaches rely on position measurements to estimate the location of unknown nodes. The sensor nodes should be equipped with special hardware to determine the position measurements, distance or bearing, from *unknown* nodes to *beacons*. Range-based approaches are the most suitable option when the indoor WSN application requires as accurate as possible position estimation, which is the case of most robotics applications.

The position measurements in range-based approaches rely on hardware incorporated by sensor nodes, such as directional or omnidirectional antennas, RF-communications, and acoustic or optical sensors. The inter-node distance is usually estimated using the propagation time of signals, e.g., the *Time of Arrival* (*TOA*) [14] between transmitter and receiver or the *Time Difference of Arrival* (*TDOA*) [30], which is based on the correlation of two or more signals with different propagation time in order to obtain accurate distance estimations. The relative angle between sensor nodes, *Angle of Arrival (AOA)*, can be estimated using an antenna array [15] or calculating the TOA difference of two transmitters/receivers separated by a fixed distance [31]. Nevertheless, the most popular inter-node measurement is the distance estimation based on RSS because most of sensor network devices are equipped with RF-based communications, and thus extra hardware is not needed. Moreover, the RSS of RF signals can be measured during communications without needing additional bandwidth or energy requirements [32]. Furthermore, RF-based position measurements permit estimating inter-node ranges through obstacles, which allows reducing the density of WSNs and avoids typical network coverage area problems of sensor networks composed of optics and acoustic devices. The problem is that RF signal strength is very unreliable because it is affected by several signal propagation effects. Range-based approaches deal with uncertainty of position measurements to provide a location estimation of unknown sensor nodes.

The most popular range-based localization approaches are probabilistic methods, which formulate localization as a Bayesian estimation problem where both sensor node state (location) and sensor measurements are modeled using probability distributions. By using this representation, sensor node can believe to be at a certain location with a certain degree. The probabilistic localization problem consists of estimating the probability density over the space of all locations. Markov Localization framework estimates this probability density [33], which captures the probabilistic foundations of many stochastic localization methods currently used. These methods have been broadly used in indoor WSNs, e.g., grid-based methods [34], some variants of particle filters [28], and probabilistic methods for cooperative localization [32]. In the robotics context, different works have used some implementation of the Bayesian Localization Framework [33] in order to estimate the robot location using both Wireless Local Area Network (WLAN) signal strength [35–37] and range readings from radio tags [38] as sensing.

The main problem of range-based approaches is that they are strongly dependent of sensor models. For that reason the procedure for obtaining such sensor models results of paramount importance. Some techniques [39] aim to learn accurate signal strength sensor models in order to make use of available indoor infrastructure, including signals detected from WLAN and RFID beacons. In the case of probabilistic methods, sensor models usually consist of normal distributions, which are determined using the central limit theorem, *i.e.*, by repeating the measurement a sufficiently large number of times under similar conditions to determine the mean and variance of a Gaussian distribution. However, practical experience suggests that these assumptions are often violated in reality, especially when we cannot reproduce the conditions of measurements or they are unknown. In the case of fuzzy approaches, sensor models consist of fuzzy sets that represent the different facets of uncertainty affecting the measurements. These fuzzy sets are adjusted approximately, normally by human-experts that would base on their experience or an expert system, depending on the vagueness, imprecision, and unreliability of position measurements. For that reason fuzzy techniques are applicable in domains where assumptions of other methods are not satisfied, e.g., when sensor model cannot be easily elicited [40]. Some examples of fuzzy logic in localization approaches are tracking in wireless networks [41], multisensor fusion of uncertain information [42,43], location information fusion in multirobot systems [44], dynamic localization using fuzzy matching patterns techniques [45], and fuzzy inference to deal with imprecision [46] or adapt some parameters [47] of other localization methods.

In this work, we propose a range-based indoor WSN localization method that aims to avoid the typical drawbacks of methods strongly dependent on signal strength calibration. In particular, the proposed method is focused on simple on-site startup and robustness.

## 3. Perception

We use a propagation model of the RF signal strength attenuation (RSS) in order to fit the sensor model used for estimating the inter-node distances. Such a model depends on several factors, such as kind of terrain, obstructions in the wave path, atmospheric conditions, and other phenomena. These factors induce the three phenomenon that cause radio signal distortions and give rise to signal fades, as well as additional signal propagation losses [48]; reflection, diffraction, and scattering. Indoor is probably the worse situation because there are multipath reflections, diffraction around sharp corners or scattering from wall, ceiling, or floor surfaces. The different models depend on environmental conditions, which usually rely on computing the median path loss for a link under a certain probability that the considered conditions will occur.

In our case, we have adopted the *shadowing propagation model* [48], which consists of two parts: path loss and variation of received power. Path loss predicts the received power mean at the distance *d*, denoted by *P_L_*(*d*), that it is calculated relative to a reference distance *d*_0_ as follows,

(1) $$\frac{P_{L}\left(d_{0}\right)}{P_{L}\left(d\right)}={\left(\frac{d}{d_{0}}\right)}^{\beta }$$

where *β* is the path loss exponent, which is empirically determined. When path loss is measured in *dB* it can be expressed as follows,

(2) $${\left[\frac{P_{L}\left(d\right)}{P_{L}\left(d_{0}\right)}\right]}_{\mathrm{dB}}=-10\cdot \beta \cdot \log\left(\frac{d}{d_{0}}\right)$$

The variation of received power is represented as a log-normal random variable, *i.e.*, a Gaussian distribution denoted by *Ψ_dB_* when it is measured in *dB*. Thus, the propagation model is represented as follows,

(3) $${\left[P_{L}\left(d\right)\right]}_{\mathrm{dB}}={\left[P_{L}\left(d_{0}\right)\right]}_{\mathrm{dB}}-10\cdot \beta \cdot \log\left(\frac{d}{d_{0}}\right)+\Psi_{\mathrm{dB}}$$

Finally, the received power is the difference between transmitted and attenuated power,

(4) $${\left[P_{r}\left(d\right)\right]}_{\mathrm{dBm}}={\left[P_{t}\left(d\right)\right]}_{\mathrm{dBm}}-{\left[P_{L}\left(d\right)\right]}_{\mathrm{dB}}$$

where *P_r_*(*d*), *P_t_*(*d*), and *P_L_*(*d*) are received, transmitted, and attenuated power respectively, *d* and *d*_0_ are the distance and the reference distance respectively, *β* is the path loss exponent, and *Ψ_dB_* is a standard normal Gaussian distribution *N*(0, *σ*).

The propagation model is customized for RF communications of the *Tmote Sky* commercial device. This device provides two indicators that can be used for elucidating the sensor model: Received Signal Strength Indication (RSSI) and Link Quality Indication (LQI). The latter is the quality parameter, or error rate, of packet reception. The inter-node distance can be estimated by RSS because all anchor nodes are configured for emitting at maximum RF power, and hence, the distance is estimated as the attenuation of signal strength relative to such a reference value.

**Figure 1.**
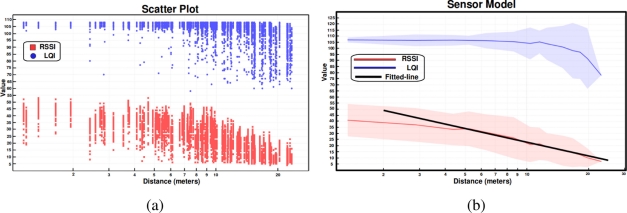
**(a)** *Tmote* indicators at different inter-node distances in an office-like environment, and **(b)** sensor model based on RF power signal strength attenuation.

Thousands of measurements are taken from different locations of an office-like indoor environment in order to fit the propagation model. By knowing the ground truth of the sensor node to estimate and the position of the *beacons* emitting at maximum power, the *Tmote* indicators can be correlated for estimating the inter-node distances. Figure 1(a) shows the *Tmote* indicator values, path loss (dBm) and LQI (dimensionless), at different transmitter-receiver distances in an office-like environment, including measurements through obstacles, such as walls and office furniture. We can observe that *Tmote* indicators are very unreliable at all distances because the uncertainty of measurements depends on several factors, such as propagation effects and environment layout.

The sensor model is obtained by fitting the RSSI values using a least squares fitting method. The fitting values are [*P_L_*(*d*_0_)]*_dB_* = 59.95, *β* = 3.72 and *d*_0_ = 1. Figure 1(b) shows the gap of the *Tmote* indicators received at different distances and the fitted sensor model based on RSSI. We have noticed that LQI indicator cannot be used for estimating distances because these values are so similar along the inter-node range. However, they can be used for filtering out measurements that do not correspond to distance estimations using RSS, e.g., distance estimations shorter than five meters that are not contained in the interval [103,110] of LQI values are rejected. We have to remark that this sensor model is approximate for a certain indoor environment, but it will be used for any office-like environment.

The statistical model permits estimating inter-node distances given the RSSI indicator of *Tmote* devices, but we can observe that these estimations are highly unreliable. For example, Figure 1(b) shows that a RSSI value of 43 corresponds to a distance of three meters according to the fitted statistical model, however, this value can correspond to any distance within the interval [1, 9] meters according to the scattering of measurements. For that reason we should include uncertainty in the sensor model in order to deal with it. How to represent and handle uncertainty of position measurements is a key point in indoor WSN localization problem. Next section presents the formalism adopted to represent and deal with uncertainty of position measurements obtained both from range estimations and from odometry.

## 4. Localization

### 4.1. The Fuzzy Approach

We define the indoor WSN localization problem as a *fuzzy estimation problem* where both the state to estimate and the position measurements are represented using fuzzy sets. Fuzzy estimation consists of determining the fuzzy density over the space of all locations. We represent location information as a fuzzy subset *μ* of the set *X* of all possible locations [49,50]. For example, *X* can be a two-dimensional space encoding the (*x, y*) position coordinates of a sensor node. For any *x* ∈ *X*, the value of *μ*(*x*) (*μ*(*x*) ∈ [0, 1]) is read as the degree of possibility that the robot is located at *x* given the available information. Total ignorance is represented by the fuzzy location *μ*(*x*) = 1 for all *x* ∈ *X*.

The *fuzzy density* or *fuzzy belief G* is defined as the density over all possible locations where the robot could be located. Thus, the localization problem can be formulated as maintaining the belief *G_t_* that represents the robot’s position at time *t*. The aim of localization is making this belief as close as possible to the real distribution of the robot’s pose. Ideally, the robot’s belief has a single peak at the true location and it is zero everywhere else. Unfortunately, uncertainty is always present in reality.

The *fuzzy density* is estimated following the typical predict-update cycle of recursive state estimators [51]. The prediction stage consists of a dilation of the *fuzzy belief G*_*t*−1_ in order to obtain the predicted *fuzzy belief* *G*′_*t*_. This operation is performed by a fuzzy dilation operator [52,53] *B* that dilates the *fuzzy belief G*_*t*−1_ in all directions in order to represent both the robot’s motion and the uncertainty in the robot’s location. In the case that we know that the sensor node is static, the fuzzy dilation is also applied to guarantee the convergence of the method and to ensure that the recursive estimator is not trapped into a local minimum. Formally, the dilation operation of *G* by *B* is denoted by *G* ⊕ *B*, and the prediction stage is defined by

(5) $${G^{\prime }}_{t}=G_{t-1}⊕B$$

which dilates the *fuzzy belief* from *G*_*t*−1_ to *G*′*_t_*. Intuitively, the result of a fuzzy dilation is a fuzzy distribution spatially expanded from *G*, where *B* represents the shape of the expansion. In our implementation, we have adopted an isotropic *B* operator which expands *G* in all directions.

**Figure 2.**
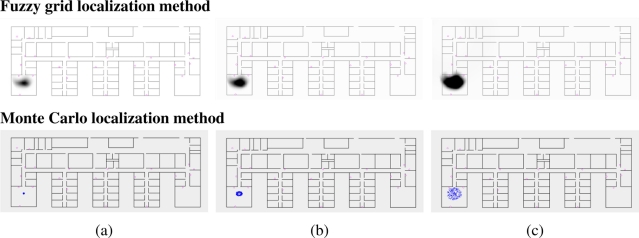
Example of *action model* (prediction phase) of the fuzzy grid (upper) and Monte Carlo (lower) localization approaches.

Figure 2 (upper) shows three examples of fuzzy grid distributions: the gray level in each cell represents the degree of possibility that the robot is located at that cell’s location (darker cells indicates higher degrees). This example shows the resulting robot’s belief *G*′_*t*+2_, shown in Figure 2(c) (upper), after two dilation operations given the robot’s belief *G_t_*, shown in Figure 2(a) (upper), at time *t*. This sequence is possible when the robot does not detect any observation during some predict-update cycles, and hence, the update stage does not modify the fuzzy robot’s belief.

The update stage consists of intersecting the predicted belief *G*′*_t_* with the beliefs induced by all observations (inter-node position measurements) at time *t*. Let *S_t_*(·|*r*) be the possibility distribution induced by the observation *r* at time *t*. In other words, *S_t_*(·|*r*) represents the possibility that the robot is located at (·), n-dimensional fuzzy state, given the position measurement *r*. The predicted fuzzy belief *G*′*_t_* is then updated by intersecting it with the fuzzy distributions *S_t_*(·|*r*_1_), *S_t_*(·|*r*_2_), . . . *S_t_*(·|*r_n_*) induced by the observations *r*_1_, *r*_2_,. . ., *r_n_* at time *t* as follows,

(6) $$G_{t}\left(\cdot \right)={G^{\prime }}_{t}\left(\cdot \right)\cap S_{t}\left(\cdot |r_{1}\right)\cap S_{t}\left(\cdot |r_{2}\right)\cap \cdots \cap S_{t}\left(\cdot |r_{n}\right)$$

where ∩ denotes a fuzzy intersection operator. There are different choices for ∩ depending on the independence assumptions made about the items being combined [54]. In our case, we have adopted the fuzzy product operator because it reinforces the effect of consonant observations. The fuzzy intersection operation satisfies the associative property, but commonly, normalization is performed after each intersection. Since fuzzy normalization is a non-associative operation, the order in which intersection operations are performed modifies the final result of the fuzzy robot’s belief.

**Figure 3.**
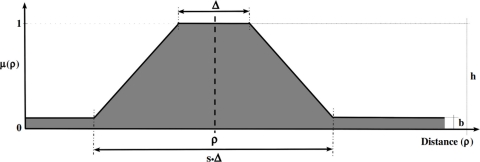
Fuzzy set representation of a position measurement observed at the distance *ρ*.

The uncertainty of the observations is represented as different intervals in the inter-node range; the sensor model of each observation is associated with a trapezoidal fuzzy set *μ*(*x, y*) = (*ρ,* Δ, *s, h, b*), shown in Figure 3 in the dimension of the inter-node distance instead of the 2*D* grid, that represents the uncertainty of the position measurement. The *ρ* parameter is the center (inter-node distance estimation), Δ is the width of the core, *s* · Δ is the width of the support, *h* is the height, and *b* is the bias. The width of the core represents a completely possible area (imprecision representation) where we assume that the robot is located. The slopes of the trapezoidal fuzzy set, width of the support excepting the width of the core, represent an area where the robot could be located (vagueness representation). The bias of the trapezoidal fuzzy set represents the area where there is a small possibility that the robot is located (unreliability representation). In our implementation, the parameters of the trapezoidal fuzzy set, representing the imprecision, vagueness, and unreliability of each observation, are adjusted depending on the inter-node distance estimation *ρ*. We only adjust the width of the core Δ and the width of the support *s* · Δ in order to represent the facets of the uncertainty mentioned above. We have followed the criterion of close observations inducing a smaller area in the fuzzy robot’s belief than the further ones, *i.e.*, instead of weighting the importance of the position measurement (using different heights in the trapezoidal fuzzy sets) depending on the inter-node distance estimation *ρ*, we model the areas where it is fully possible, possible, and unlikely that the robot is located.

**Figure 4.**
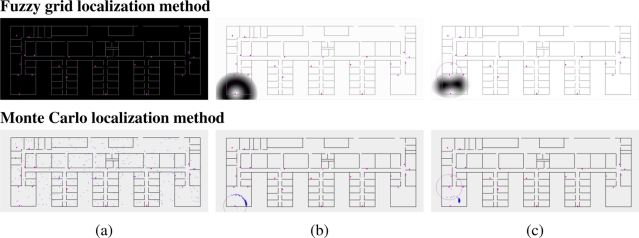
Example of *sensor model* and update phase of the fuzzy grid (upper) and Monte Carlo (lower) localization approaches.

Figure 4 (upper) shows an example of the fuzzy robot’s belief representation and the fuzzy beliefs induced by the observations (position measurements). We can observe how the grid-based representation of the fuzzy belief is able to represent both total ignorance and multiple possible locations; the robot’s location is initially ignored, and hence, all positions are fully possible, shown in Figure 4(a) (upper), after two updates there are a couple of possible robot’s positions, shown in Figure 4(c) (upper), represented by two clouds in a room of the office-like environment. Figure 4(b) (upper) shows the fuzzy belief induced by an observation, where we can observe the different intervals encoding the fully possible positions (imprecision), the possible positions (vagueness), and the unlikely positions (unreliability).

### 4.2. The Reference Method

The reference method is a variant of Monte Carlo localization approach [55], in which the probability density is represented by maintaining a set of samples that are randomly drawn from it. Such a variant uses a hybrid representation of the probability density to reduce the computational cost. The pose probability is factorized as a distribution over a continuous set of angles and continuous translational coordinates; the distribution over poses (x,y,*θ*) is first generically decomposed into the product *P* (*x, y, θ*)=*P* (*θ*) · *P* (*x, y*|*θ*)=∑*_i_ P* (*θ_i_*) · *P* (*x, y*|*θ_i_*), which is a kind of Rao-Blackwellization of the state space [56,57]. The distribution *P* (*θ*) is modeled as a discrete set of weighted samples *θ_i_*, and the conditional likelihood *P* (*x, y*|*θ*) as simple two-dimensional Gaussian. This approach has the advantage of combining discrete Markov updates for the orientation with Kalman filter updates for the translational degrees of freedom. Note that though there is not bearing information, due to range-only measurements, the orientation can be estimated when the robot is in motion. Besides, the simulation of omnidirectional random noise is facilitated including an orientation hypothesis into each sample, even when sensor node is static.

As in the case of the proposed localization approach, the Monte Carlo method follows the typical predict-update cycle of recursive state estimators. The prediction stage consists of the simulation of the motion of each sample including random noise in order to improve the convergence. Figure 2 (lower) shows three examples of probabilistic distributions: the set of samples represents the positions where it is probably located the robot. The example shows the resulting robot’s belief, shown in Figure 2(c) (lower), after two update stages given the probability distribution of the robot, shown in Figure 2(a) (lower), at time *t*. The sequence is only possible if the robot does not detect any observation during some predict-update cycles. The update stage consists of a product operation and a resampling; the product operation is performed between each sample of the predicted probabilistic belief and the sample induced by each observation (inter-node position measurements) at time *t*, whereas the resampling aims to remove the samples with a low probability after the product operation. Figure 4 (lower) shows an example of the probabilistic robot’s belief and the beliefs induced by the observations (position measurements). The representation used in the Monte Carlo method is able to represent both total ignorance and multiple possible locations depending on the number of samples used to represent the robot’s belief; the total ignorance about the robot’s position at time *t* is represented by a set of samples randomly distributed along the environment, shown in Figure 4(a) (lower), and after two update stages there are a concentration of samples in one of the two locations obtained by the proposed method, shown in Figure 4(c) (lower), although there are also samples in the other possibility.

## 5. Experimental Validation

This section presents the experimental validation of the proposed approach using real data. This is a key point because one of most important reasons of failure in localization methods is the unknowledge about sources of noise, which is usually left out when localization approaches are evaluated using simulations. Besides, an experimental comparison with one of most popular stochastic localization methods is performed in order to evaluate the differences between them.

The battery of tests consists of kidnapping and tracking experiments using both the proposed approach and a stochastic reference method. In the robotics context, the kidnapping problem consists of positioning the robot at a location, and all of a sudden it is transferred or “kidnapped” to other location without the robot being aware of this. The kidnapping experiment is useful in order to evaluate the robustness of localization methods in different situations, such as false positive observations and recovery from failures. The tracking experiment consists of estimating the robot’s location when it is in motion. We have to remark that motion is an important source of uncertainty when the robot navigates because RSS is affected by bearing modifications of antenna. Thus, the experiments evaluate usual situations (like tracking) and unusual situations (like kidnapping or recovering from failure).

**Figure 5.**
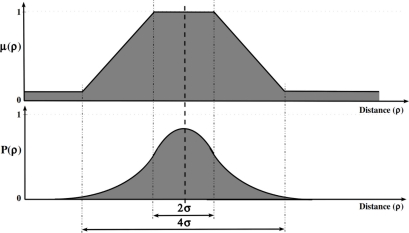
Sensor models used to perform the comparison experiments; correspondence between (upper) fuzzy set and (lower) stochastic Gaussian distribution.

All the experiments are performed using both the proposed approach and the reference method in order to make the comparison between them. In order to make this comparison as fair as possible, we have used similar sensor and action models. How to do this, however, is not obvious since fuzzy and probabilistic techniques are semantically different: we interpret fuzzy sets to represent degrees of possibilities, while probabilities are more naturally interpreted in terms of stochastic events [23]. Moreover, stochastic methods need sensor models based on frequencies, and hence, the probability function that models the sensor should be experimentally obtained, whereas methods based on fuzzy logic make use of qualitative sensor models. We have ignored these semantic differences, and we have used probabilistic sensor models that directly reflect the fuzzy ones. Thus, the stochastic sensor model is represented by a two-dimensional Gaussian function, whose parameters are chosen so that the core and the support of the fuzzy model correspond to two and four standard deviations of the stochastic Gaussian function, respectively. Figure 5 shows the correspondence between the proposed method and the reference one.

### 5.1. Experimental Setup

**Figure 6.**
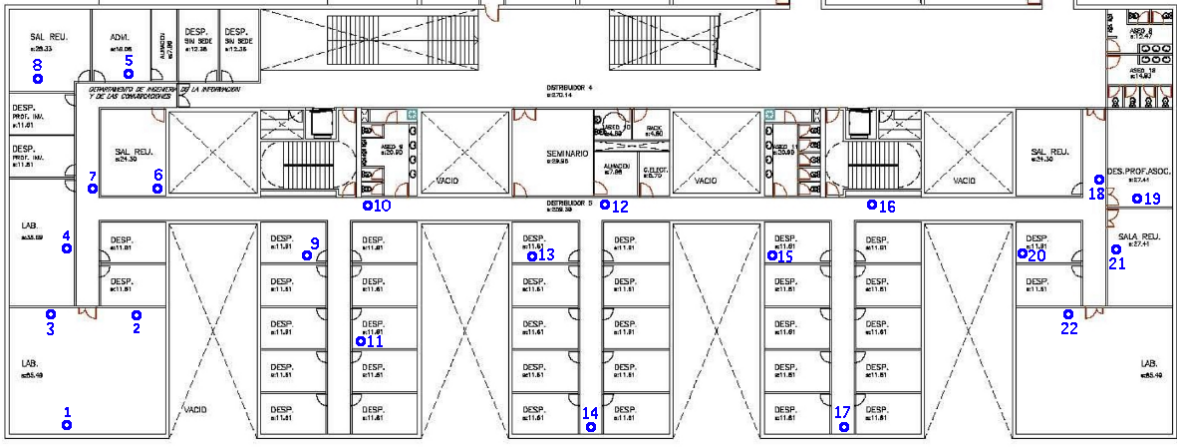
The environment and the deployment of the beacons.

The proposed method is evaluated using an indoor WSN composed of several RF beacons, *Tmote Sky* devices, distributed along an office-like environment. Figure 6 shows the floor plant layout and the deployment of the beacons. We can observe that beacon density is not so high, and hence, the proposed localization method is evaluated in this unfavorable situation.

**Figure 7.**
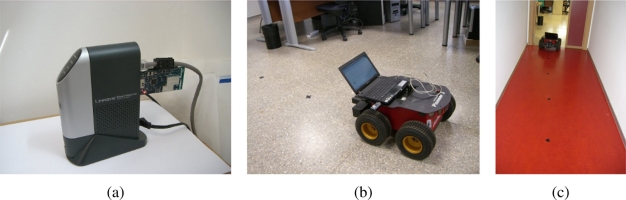
Experimental setup: **(a)** WSN beacon installed in a NAS device, **(b)** mobile robotic platform, and **(c)** the robot performing a tracking experiment.

The sensor node used by the robot is part of the WSN, in particular a commercial *Tmote Sky* device. This platform makes use of an integrated omnidirectional on-board antenna providing up to 125 meters range. Besides, it incorporates an *IEEE* 802.15.4 Chipcon Wireless Transceiver which is able to transmit 250 *kbps* at 2.4 *GHz* frequency with an output power between −25 and 0 dBm. The anchor nodes are installed in the Network-Attached Storage (NAS) devices distributed along the scenario; in particular, the beacons are installed through the USB port of the *Linksys NSLU2* devices, shown in Figure 7(a), distributed along the office-like environment.

The experiments are performed using a four wheel drive robotic platform, *Pioneer 3-AT* shown in Figure 7(b), equipped with a laptop on the top, which drives the vehicle using the serial port and communicates with WSN through a *Tmote Sky* device. All WSN devices are synchronized in order to avoid emitting packets at once, and thus inducing interferences. Besides, all beacons are configured for emitting packets at maximum RF power in order to use the sensor model elicited above.

The experiments consist of driving the robot between known positions, which permits estimating the ground truth and calculates the position error. While the robot navigates between known locations, shown in Figure 7(c), it estimates its position using the communication packets received from beacons. The operator indicates when the robot has reached a known position and thus ground truth is estimated by dead-reckoning using odometry from known locations. Since known locations are relatively close we assume that position error due to odometry is not significant, and hence such position estimations can be used as ground truth.

### 5.2. Kidnapping Experiment

The kidnapping experiment consists of positioning a sensor node at whatever initial location estimating its pose using the messages received from beacons, and after some seconds it is transferred to other location without the sensor node being aware of this. This process is repeated several, almost hundred, times. The locations where the sensor node is transferred are known, and the localization techniques are activated when the sensor node is located at a new pose and deactivated when the sensor node is “kidnapped”. The aim of deactivating the localization process, while the sensor node is transferred to a new location, is to simulate an instantaneous transfer which should be interpreted and handled by the localization method, normally as recovery from failure. These experiments permit us to evaluate the convergence and robustness of the localization methods, and the quality of the position estimation. Note that environment information is obviated in these experiments.

**Figure 8.**
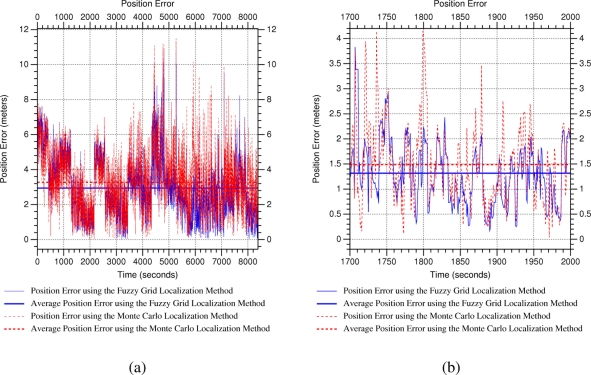
Position error in kidnapping experiment: **(a)** long and **(b)** short series. Note that the peaks of position error correspond to the instant after the sensor node is kidnapped.

Figure 8(a) shows the position error along the whole experiment. We can observe that the average of the position error is almost the same, around three meters, using both methods. Note that the average position error includes position error during convergence time, *i.e.*, since the sensor node is transferred to a new location until the localization approach converges to such a position. Besides, the localization approaches are evaluated from almost all possible positions in the environment and, given the low density of WSN beacons in the scenario, there are some areas where received information do not permit locating properly the sensor node. Figure 8(b) shows the position error during a short period in which beacon layout permits estimating the sensor node location from the poses where it is transferred. We can observe position error peaks when the sensor node is transferred to a new location, and how position error is reduced when the localization approach converges. We obtain position errors of less than one meter for both localization approaches when they converge from poses receiving information from enough beacons. We have to remark that the estimated sensor node is static in these experiments, and that motion is an important source of uncertainty in WSNs, which is evaluated in next section.

### 5.3. Tracking Experiments

The tracking experiments consist of estimating the location of a sensor node installed on the robot platform while it navigates through a route defined by known way-points. When the mobile robot reaches these way-points, an operator notifies it by sending a packet to the robot in order to indicate the ground truth and to calculate the position error. The experiments evaluate two unfavorable situations when there are long corridors in indoor environments: crossing an intersection with a long corridor and navigating through a long corridor. The multipath reflection effects and the line-of-view between emitter and receiver in long corridors induce very noisy RSS measurements with respect to the sensor model elicited above. This is because of such a sensor model considers average signal attenuation, which includes walls and other obstacles; in particular, multipath reflections induce further distance estimation due to higher RSS attenuation, while free line-of-view between emitter and receiver induces closer distance estimation due to lower RSS attenuation.

**Figure 9.**
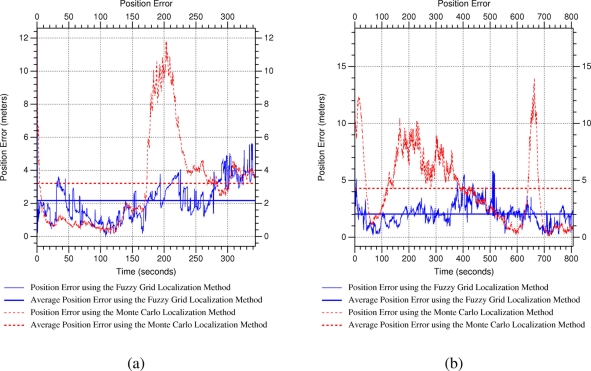
Position error in tracking experiment: **(a)** the robot crossing an intersection with a long corridor and **(b)** the robot navigating through a long corridor.

Figure 9(a) shows the position error when the mobile robot is crossing an intersection with a long corridor. We can observe that the proposed localization method provides better estimations than Monte Carlo when the position measurements are very unreliable, *i.e.*, when the robot is located at the cross-road, whereas it provides similar position estimations when the position measurements are relatively accurate. Figure 9(b) shows the position error when the mobile robot is navigating through a long corridor. In contrast to previous experiment, position measurements are really unreliable during the whole experiment. We can observe that the proposed localization approach also provides better position estimation than the reference method. Besides, average position error using the proposed localization approach is about two meters in this unfavorable situation, which supposes the double of accuracy than using the reference method.

The reasons for providing the proposed method better position estimations in highly uncertain situations are: the representation of approximate location information and specially the ability of fuzzy logic for addressing the fusion information problem. The reference method makes use of a classical weighted average fusion of the different sources of location information, whereas the proposed method permits maintaining the information making a decision about the sources being combined, and typically obtaining a consensus between the different sources of location information.

**Figure 10.**
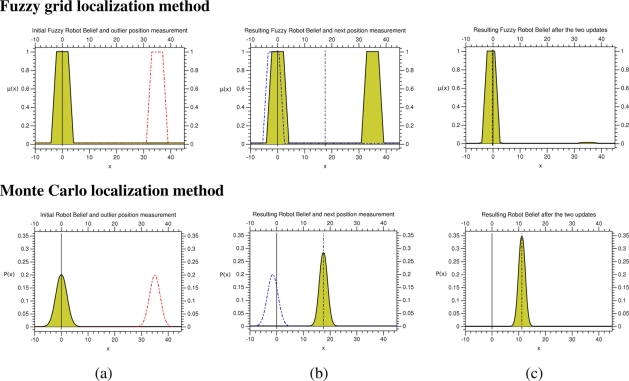
Example of location information fusion using the proposed and the reference localization methods.

Figure 10 shows a numerical example of the proposed approach and the reference method, which aims to show what is happening in the long corridor of the tracking experiments. The example is shown in one dimension for graphical clarity. Initially, the robot is located in the origin of the dimension, represented by a continuous line, and the fuzzy robot’s belief has a certain distribution that directly reflects the stochastic one following the criterion adopted in the previous experiments. The robot then detects a wrong observation in a long corridor, shown in Figure 10(a), due to the free line-of-view between the emitter and receiver, which induces closer distance estimation from the beacon due to lower RSS attenuation. We can observe how the fuzzy approach maintains the representation of both sources of information as consequence of the fuzzy intersection and the fuzzy normalization, whereas the stochastic method makes a weighted average fusion. The Center of Gravity (CoG) of the resulting fuzzy robot’s belief, represented by the dotted line, matches up to the mean of the resulting probabilistic robot’s belief, shown in Figure 10(b). However, the former distribution is able to maintain the information of the “wrong” measurement in order to further check its cause (outlier, failure, kidnapping, *etc.*). Finally, the robot detects a proper observation; in the case of the fuzzy approach, the fuzzy intersection and normalization induce a fuzzy robot’s belief covering the real position of the robot, whereas in the case of the probabilistic method the weighted average fusion provides a probabilistic distribution farther away from the real position of the robot. Figure 10(c) shows how the CoG of the resulting fuzzy robot’s belief is very close to the real robot’s position, while the mean of the probabilistic distribution is farther away from the real robot’s position.

Some probabilistic localization methods perform different tests in order to check if the measurement is an outlier given the probabilistic distribution of the robot’s belief, and then avoiding the problem presented in the numerical example. For example, the Extended Kalman Filter (EKF) makes use of the distance of Mahalanobis in order to compare the innovation of the state to estimate and the covariance associated to such an innovation considering the measurement. This comparison is used to filter out outliers, *i.e.*, measurements that are not coherent with the robot’s position considering the covariance of the robot’s belief. However, this kind of tests compromises the localization approach when kidnapping or recovering from failure, *i.e.*, it is then only able to handle the tracking (local localization) problem.

## 6. Conclusions

We have presented the development and experimental evaluation of a fuzzy localization framework for addressing the indoor WSN localization problem in general, and the indoor WSN localization of a mobile robot using uncertain inter-node range measurements in particular. The proposed approach is focused on simple setting and robustness; simple setting is achieved by adjusting the fuzzy sets that represent location uncertainty in position measurements, while uncertainty management permits estimating a consensus between the different sources of information, instead of classical weighted average fusion, which improves the robustness of the position estimation. The on-site startup by simply tuning the approximate sensor models supposes an important advantage with respect to popular WSN localization approaches based on signal strength calibration because the setting of these systems is complex and time consuming. The experimental evaluation of the proposed method confirms that the fuzzy localization approach is able to solve the typical local (tracking) and global (recovery from failure and ignorance of initial location) localization problems in robotics. Finally, we have demonstrated that the proposed approach results in feasible and robust low-density WSNs. For all these reasons, we can state that the proposed approach can be simple and quickly configured in indoors providing accurate position estimations, even when high uncertainty in the position measurements.
